# Energy Transfer in Dendritic Systems Having Pyrene Peripheral Groups as Donors and Different Acceptor Groups

**DOI:** 10.3390/polym10101062

**Published:** 2018-09-25

**Authors:** Pasquale Porcu, Mireille Vonlanthen, Andrea Ruiu, Israel González-Méndez, Ernesto Rivera

**Affiliations:** Instituto de Investigaciones en Materiales, Universidad Nacional Autónoma de México, Circuito Exterior Ciudad Universitaria, Ciudad de México C.P. 04510, Mexico; paskpo89@gmail.com (P.P.); andrea1.ruiu@gmail.com (A.R.); israel__025@hotmail.com (I.G.-M.)

**Keywords:** pyrene, dendritic molecules, energy transfer

## Abstract

In this feature article, a specific overview of resonance energy transfer (FRET) in dendritic molecules was performed. We focused mainly on constructs bearing peripheral pyrene groups as donor moieties using different acceptor groups, such as porphyrin, fullerene C_60_, ruthenium-bipyridine complexes, and cyclen-core. We have studied the effect of all the different donor-acceptor pairs in the energy transfer efficiency (FRET). In all cases, high FRET efficiency values were observed.

## 1. Introduction

Since their discovery at the end of the 80’s, the scientific community has been very interested in dendrimers due to their well-defined nanostructures that confer them outstanding chemical and physical properties [1,2,3]. The special attention accorded to dendrimers is essentially due to their potential applications in diverse fields such as in catalysis, drug delivery and photoactive materials [4,5,6,7,8,9,10]. The investigation of the photophysical properties of these molecules is very attractive and depends in large measure on the architecture of these perfectly branched macromolecules. The incorporation of photoactive units into dendrimers can be achieved by attaching them covalently or noncovalently in three possible locations: periphery, branches, or core. Many photoactive chromophores have been linked to dendrimers through covalent bonds to produce highly luminescent materials [11,12].

Depending on the selected photoactive molecules and their position in the dendritic construct, different phenomena, such as fluorescence resonance energy transfer (FRET), excimers, and charge transfer (CT) can take place [13]. The study of those processes is very important, since it helps us to make innovations to improve the efficiency of existing photovoltaic devices [13,14,15,16,17]. With that aim, many scientists have focused their research on the design and study of various dendritic molecules introducing different chromophores with particular optical and photophysical properties [18,19,20,21]. 

Among the available chromophores, pyrene is considered the most used fluorescent dye in the study of labelled polymers [22], and there are some reviews covering the most important aspects of its fluorescent properties [23,24,25,26,27,28,29,30,31]. The use of pyrene as a fluorescent label to study polymers of different lengths and architectures is prevalent because of its unique photophysical behavior, mainly its tendency to form excimers. Pyrene moieties have been attached to numerous macromolecules [32,33] in order to investigate micellization, polymer, and dendrimer dynamics [34], and for the development of new π-conjugated polymers and oligomers with pyrene units [35,36]. This phenomenon has been also observed in dendritic structures bearing pyrene previously reported [37,38,39]. Numerous dendritic architectures with photoactive groups have been reported so far, but there are only a few examples where pyrene has been covalently attached jointly with the presence of an acceptor group to give an efficient energy transfer (FRET) for light-harvesting purposes. 

### Optical and Photophysical Features of Pyrene

Pyrene as a chromophore has outstanding optical properties, like elevated quantum yield, long fluorescence lifetime, and excimer emission, depending on its local concentration [22]. That is why it is often used as a probe to test dynamic processes of polymers in solution that take place in the pyrene lifetime regime [22,23,24,25,26,27]. Other interesting features of pyrene are its potential use in dyads for solar energy conversion and as its use in the exfoliation of carbon nanotubes (CNT) and graphene [40].

**Figure 1:**
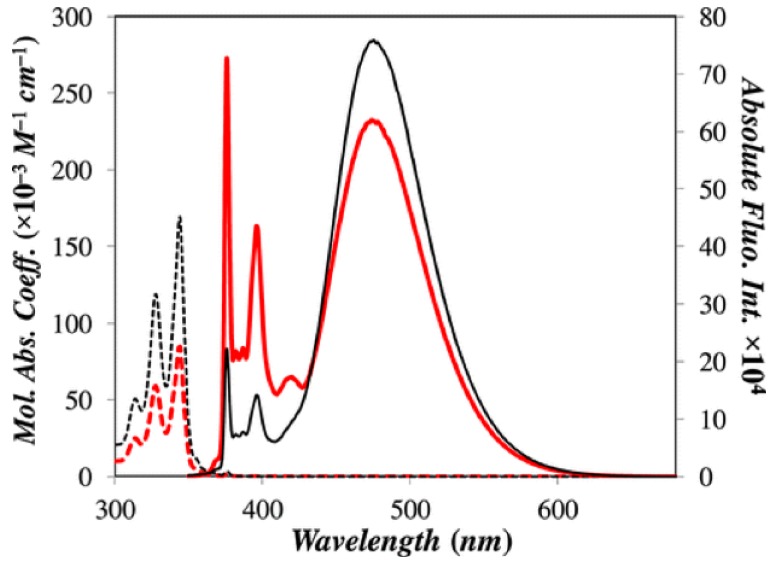
Absorption (dashed lines) and fluorescence (solid lines) spectra of first (Py_2_-G1OH; red line) and second (Py_4_-G2OH; black line) generations pyrene-labelled dendrons in THF. [Py] = 1.25 × 10^–6^ M; λ_ex_ = 344 nm. Reprinted with permission from (Zaragoza-Galán, G., Fowler, M.A., Duhamel, J., Rein, R., Solladié, N., & Rivera, E. (2012). Synthesis and Characterization of Novel Pyrene-Dendronized Porphyrins Exhibiting Efficient Fluorescence Resonance Energy Transfer: Optical and Photophysical Properties. *Langmuir*, *28*(30), 11195–11205.). Copyright (2012) American Chemical Society [47].

We have investigated the photophysical properties of various polymers and compounds bearing pyrene [36]. Pyrene has been also incorporated into various macromolecules with the aim to develop novel photoactive materials [35,36,41,42,43,44,45,46]. Furthermore, we have designed a Fréchet-type dendron containing an increasing number of pyrene moieties with the generation number. It these constructs, pyrene acts as donor in a dual way, from the monomer or excimer emission, depending on the structure of the dendron. The absorption and emission spectra of the dendron of the first generation (Py_2_-G1OH) as well as of the dendron of the second generation (Py_4_-G2OH) is shown in Figure 1.

Based on the obtained results, we designed dendritic systems bearing different donor-acceptor pairs that behave as molecular antennae, keeping pyrene as the donor group, such as pyrene-porphyrin [47], pyrene-fullerene [48], pyrene-ruthenium bipyridine complexes [49], and the pyrene-cyclen core [50].

## 2. Dendritic Molecules Bearing Peripheral Pyrene Groups as Donors and a Porphyrin as an Acceptor

Porphyrins belong to an important category of fluorophores, deeply investigated in materials science [51,52,53]. The development of novel porphyrins and multiporphyrinic systems attracted our attention because of their potential applications in various fields such as nonlinear optics (NLO), to photon absorption, molecular wires, and catalysis [54,55,56,57]. Porphyrins have been covalently functionalized with various electro- and photoactive groups with the aim to tune their opto-electronic and photophysical properties. In particular, we can modify the donor-acceptor character of porphyrins by linking them covalently with photoactive groups or by coordinating them with different metal ions [58,59,60].

Even though the synthesis and the study of the optical and photophysical properties of many porphyrinic compounds attached to electro- and photoactive groups like for example fullerene C_60_ [58] and anthracene [59] or other functionalized porphyrins [60] have been published, there are only a few reports about porphyrin–pyrene systems [61]. Moreover, none of these reports is an investigation about light harvesting or the energy transfer phenomenon. Provided that monomer and excimer emissions of the pyrene [22] are partially superimposed with the Soret absorption band of porphyrins (λ = 419 nm for tetraphenyl porphyrin (TPP) and λ = 423 nm for its Zn-metallated derivative (ZnTPP)) [62,63,64], we can expect a very efficient fluorescence resonance energy transfer (FRET) in structures labelled with these two chromophores. Therefore, no less than three different photophysical processes can take place in these structures at the same time. Firstly, an excimer can be generated by the pyrenyl pendant groups; secondly a FRET phenomenon can occur from an excited pyrene unit or an excimer to the porphyrin, respectively. Provided that dealing with a single photophysical process taking place in an intramolecular way in a photoactive dendritic molecule is problematical to study, dealing with three simultaneous processes is a real photophysical challenge. Fortunately, the involved photophysical processes depends in large measure on controllable molecular parameters that can be modified in order to selectively affect only one photophysical process and allow a reasonable characterization of all photophysical processes. Definitely, pyrene excimer formation and FRET are significantly dependent on the pyrene content and the donor (pyrene monomer and excimer)-acceptor (porphyrin) distance.

**Figure 2:**
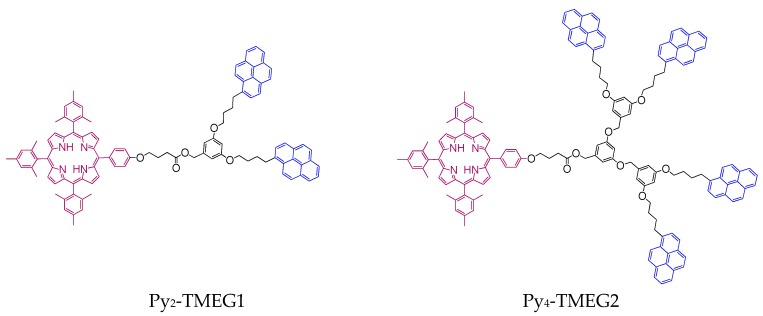
Structure of the dendronized porphyrins containing first-generation and second-generation Fréchet-type dendrons [47].

These features were seriously considered to develop two series of dendritic molecules labelled with pyrene and porphyrin. First- and second-generation poly(aryl ether) dendrons totally functionalized in the periphery with 1-pyrenebutyl units were prepared to take advantage of the elevated excimer emission that happens with increasing the generation of the dendritic constructs containing pyrene [34,37]. Furthermore, a porphyrin unit was attached to the focal point of the pyrene-labelled dendrons through a flexible spacer. Given that in a dendron the distance from the periphery and the focal point augments at higher generations, growing the generation of the pyrene-labelled dendrons from 1 to 2 augmented the distance between pyrene units and porphyrin, thereby diminishing the efficiency of FRET. Thus, growing the generation of the dendron employed in these dendritic structures increased excimer formation thereby decreasing the FRET efficiency. The structures of these dendritic molecules are illustrated in Figure 2. The synthesis of the dendronized porphyrins as well as the effect that the structural changes induce in the optical and photophysical processes has been studied in detail [47].

The synthesis and characterization of this constructs has been reported by our research group [47]. These characterization of the molecules was carried out by FT-IR, ^1^H NMR, ^13^C NMR, UV–VIS spectroscopy, and MALDI-TOF MS [47]. 

**Figure 3:**
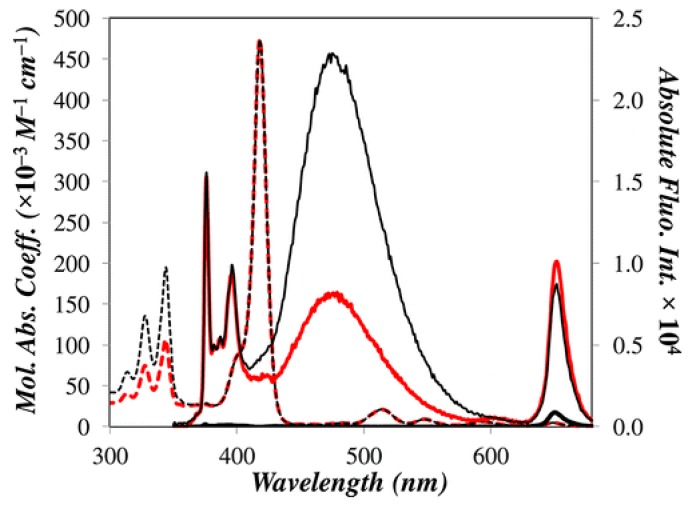
Absorption (dashed line) and fluorescence (solid line) spectra of Py_2_-TMEG1 (red line), Py_4_-TMEG2 (thin black line), and fluorescence spectrum of TME (porphyrin precursor without pyrene units) (thick black line with a low fluorescence intensity at 651 nm) in THF. [Py_2_-TMEG1] = 5.1 × 10^–7^ M; [Py_4_-TMEG2] = 2.6 × 10^–7^ M; and [TME] = 2.5 × 10^–6^ M; λ_ex_ = 344 nm. Reprinted with permission from (Zaragoza-Galán, G., Fowler, M.A., Duhamel, J., Rein, R., Solladié, N., & Rivera, E. (2012). Synthesis and Characterization of Novel Pyrene-Dendronized Porphyrins Exhibiting Efficient Fluorescence Resonance Energy Transfer: Optical and Photophysical Properties. *Langmuir*, *28*(30), 11195–11205.). Copyright (2012) American Chemical Society [47].

Absorption spectra of the dendronized porphyrins (Py_2_-TMEG1 and Py_4_-TMEG2) are illustrated in Figure 3. Py_2_-TMEG1 and Py_4_-TMEG2 exhibited the maximun absorption band at λ = 344 nm, arising from the S_0_ → S_2_ transition of pyrene, followed by the Soret band of the porphyrin at 416 nm (λ = 414 nm for Py_2_-TMEG1, and λ = 418 nm for Py_4_-TMEG2). The porphyrin unit exhibit also four Q bands, which appear in the range between 450 and 700 nm. Since the absorption spectra of the obtained dendronized porphyrins are the sum of the absorption spectra of their individual precursors having pyrene and porphyrin elements, we can affirm that there is no interaction between pyrene and porphyrin in the ground state [47]. 

Fluorescence spectra of Py_2_-TMEG1 and Py_4_-TMEG2 were recorded exciting at 344 nm in THF solution at room temperature (Figure 3). The obtained spectra were showing two emission bands at 376 nm and 476 nm corresponding to the monomer and excimer bands of pyrene, respectively, that are comparable with the emission bands of the precursor pyrene-labelled dendrons. Nevertheless, we can also observe a new band at 651 nm due to the porphyrin emission. The ratios between the excimer intensity/monomer intensity (I_E_/I_M_) were calculated for Py_2_-TMEG1 and Py_4_-TMEG2. The obtained values were 0.58 and 1.46, respectively. Those values were much lower than the values obtained for the pyrene dendron precursors Py_2_-G1OH and Py_4_-G1OH (0.69 and 3.05, respectively).

Time-resolved fluorescence experiments revealed that the pyrene fluorescence quenching happens with an energy transfer rate constant value of 2.3 and 1.8 × 10^9^ s^–1^ for Py_2_-TMEG1 and Py_4_-TMEG2, respectively. Such rate constants are the proof of a highly efficient quenching mechanism. Regardless the I_E_/I_M_ ratio, a more than 30-fold decrease in fluorescence intensity can be seen when the dendrons and the porphyrin are covalently attached, exhibiting a very efficient FRET from an excited pyrene monomer or excimer to the porphyrin. Indeed, pyrene fluorescence in the porphyrinic construct Py_2_-TMEG1 and Py_4_-TMEG2 suffer a quantitative quenching (99% and 97%, respectively) as shown in Table 1.

The fluorescence band observed at 651 nm, exciting at 344 nm, comes from the porphyrin unit of compounds Py_2_-TMEG1 and Py_4_-TMEG2. This emission is more intense than that observed after direct excitation of TME (precursor porphyrin without pyrene) in solution (Figure 3). The donor (pyrene monomer and excimer) showed a remarkable decrease in fluorescence intensity while the acceptor (porphyrin) exhibited an enhancement in fluorescence intensity, which is a clear indication that FRET is occurring in the pyrene–porphyrin dendritic constructs Py_2_-TMEG1 and Py_4_-TMEG2. E_FRET_ values and quantum yields are summarized in Table 1.

In function of the distance pyrene-porphyrin (*d_Py_*_−*Por*_), we can expect a decrease in FRET efficiency in Py_4_-TMEG2, with respect to Py_2_-TMEG1. Nevertheless, if the increase of the distance *d_Py_*_−*Por*_ is not enough to overcome the Förster radius (R_0_), this change in the FRET efficiency will not be observed. As a result, very high FRET efficiencies were observed for both generations of our dendrimer leading to the conclusion that the pyrene and porphyrin units in our constructs are located well within the R_0_ value (5.2 nm) determined for this pair of chromophores. This assumption was confirmed by molecular mechanics calculations to estimate the longest possible pyrene-porphyrin distance in a stretched conformation of the molecules [47]. It is not realistic to expect that the dendritic molecules remain stretched in solution since they tend to adopt a coiled conformation. The donor-acceptor distance in these constructs was found to be 3.0 and 3.5 nm for Py_2_-TMEG1 and Py_4_-TMEG2, respectively, well under the 5.2 nm R_0_ value, showing that efficient FRET occur in these dendritic molecules (Table 2).

We can observe that FRET efficiency is not affected by the quantity of excimer emission arising from the pyrene-containing dendrons since the FRET efficiency is not affected by the amount of excimer formed in the pyrene dendron precursors. This fact reveals that excimer formation would takes place after the FRET process occurs from the monomer emission to the porphyrin acceptor and that it does not have a contribution to the whole FRET process. Time-resolved fluorescence experiments showed that FRET from excited pyrene monomer to the porphyrin core take place more than 10 times faster than the formation of pyrene excimers in the pyrene dendrons. From these results, we can affirm that FRET from the excited pyrene monomer to the porphyrin is very efficient and occurs before the formation of the excimer. 

**Figure 4:**
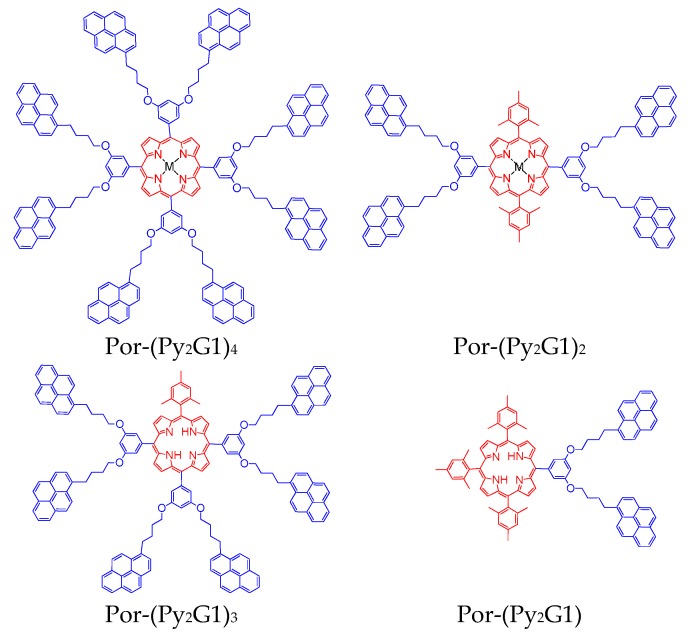
Structure of the dendrimers bearing first-generation pyrene-labelled Fréchet-type dendrons and a porphyrin core [66].

A second series of dendrimers bearing first-generation Fréchet-type pyrene-labelled dendrons and a porphyrin as the core was synthesized and characterized by our group [66]. The obtained free base porphyrins were further metallated with Zn. The structure of the obtained porphyrins was characterized by 1H NMR spectra and confirmed by MALDI-TOF mass spectrometry [66]. The electrochemical properties and charge transfer character of such systems has been also studied [67]. The structure of this series of dendrimers is illustrated in Figure 4. 

**Figure 5:**
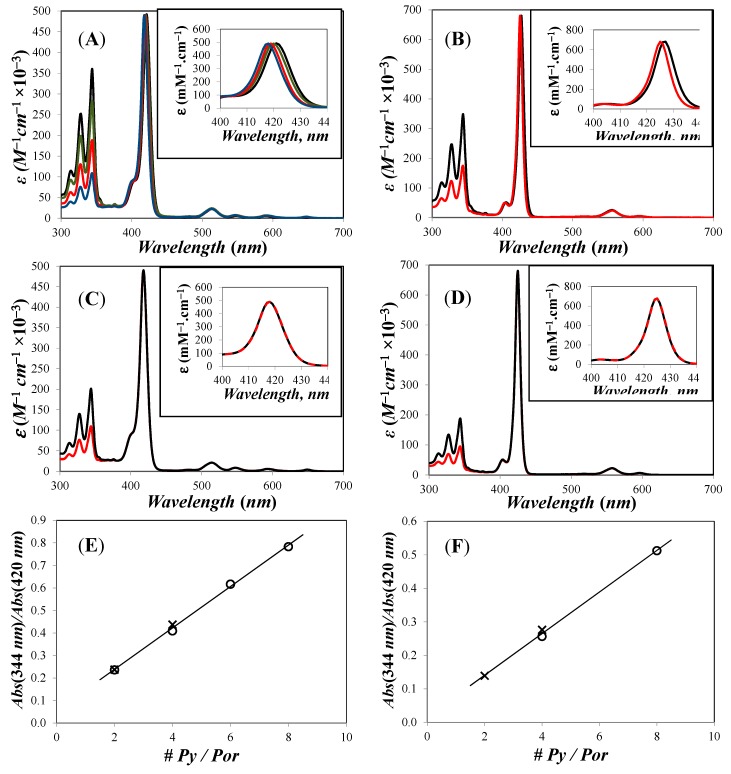
Absorption spectra of (**A**) (black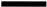) Por-(Py_2_G1)_4_, (green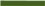)Por-(Py_2_G1)_3_, (red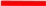) Por-(Py_2_G1)_2_, and (blue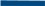) Por-(Py_2_G1)_1_; (**B**) (black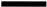) Zn-Por-(Py_2_G1)_4_ and (red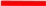) Zn-Por-(Py_2_G1)_2_; (**C**) (black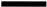) Py_4_-TMEG2 and (red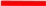) Py_2_-TMEG1; (**D**) (black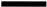) Zn-Py_4_-TMEG2 and (red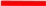) Zn-Py_2_-TMEG1; (**E**,**F**) plot of *Abs*(344 nm)/*Abs*(Soret) versus the number of pyrenyl units per porphyrinic construct for the free and metallated porphyrins, respectively. Reprinted with permission from (Zaragoza-Galán, G., Fowler, M., Rein, R., Solladié, N., Duhamel, J., & Rivera, E. (2014). Fluorescence Resonance Energy Transfer in Partially and Fully Labelled Pyrene Dendronized Porphyrins Studied with Model Free Analysis. *The Journal of Physical Chemistry C*, *118*(16), 8280–8294). Copyright (2014) American Chemical Society [66].

The absorption spectra of the porphyrinic dendrimers in THF solution are shown in Figure 5. In the absorption spectra of these compounds, we can see the typical S_0_ → S_2_ absorption band of the pyrene group centered 344 nm, as well as the Soret band of the porphyrin (free base or metalated), which appear at about 420 and 426 nm, respectively. Usually, the free porphyrin derivatives exhibit four Q bands situated between 513 and 647 nm, whereas the zinc metallated porphyrins show only two Q bands in the same UV–VIS region. 

A slight shift of the absorption band of the porphyrin moiety was observed in the series of the porphyrinic dendrimers in function of the amount of mesityl groups present in the construct. The Soret band of the free porphyrins shifted from 421 to 420, 419, and 418 nm when going from Por-(Py_2_G1)_4_ to Por-(Py_2_G1)_3_, Por-(Py_2_G1)_2_, and Por-(Py_2_G1). Likewise, the Soret band of the metalated porphyrins shifted from 427 to 425 nm when going from Zn-Por-(Py_2_G1)_4_ to Zn-Por-(Py_2_G1)_2_.

The absorption spectra of the free-base dendronized porphyrins Py_2_-TMEG1 and Py_4_-TMEG2 resulted to be the sum of the absorption spectra of 1-pyrenebutanol and porphyrin, so that we can realize that negligible to no electronic interactions occur between both chromophores.

**Figure 6:**
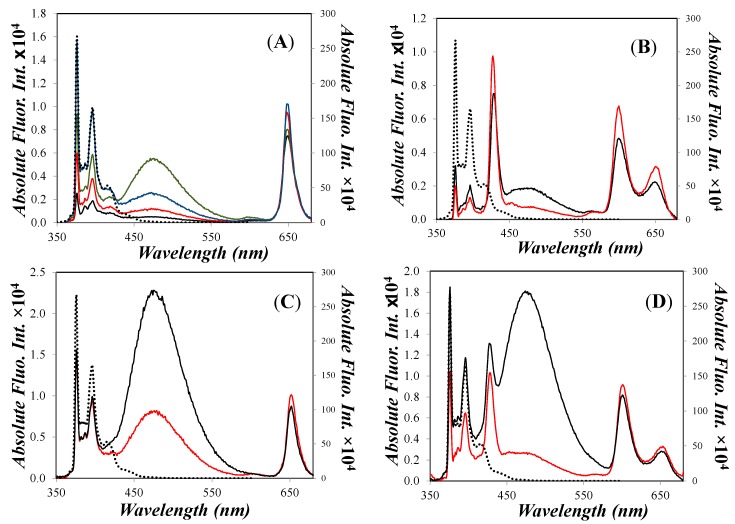
Spectra of the absolute fluorescence of (**A**) (black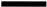) Por-(Py_2_G1)_4_, (green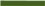) Por-(Py_2_G1)_3_, (red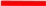) Por-(Py_2_G1)_2_, and (blue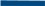) Por-(Py_2_G1)_1_; (**B**) (black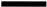) Zn-Por-(Py_2_G1)_4_ and (red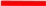) Zn-Por-(Py_2_G1)_2_; (**C**) (black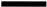) Py_4_-TMEG2 and (red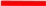) Py_2_-TMEG1; (**D**) (black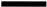) Zn-Py_4_-TMEG2 and (red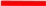) Zn-Py_2_-TMEG1; The absolute fluorescence spectrum of 1-pyrenebutanol (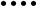) is shown in all figures with its intensity reported on the secondary axis on the right hand side. Reprinted with permission from (Zaragoza-Galán, G., Fowler, M., Rein, R., Solladié, N., Duhamel, J., & Rivera, E. (2014). Fluorescence Resonance Energy Transfer in Partially and Fully Labelled Pyrene Dendronized Porphyrins Studied with Model Free Analysis. *The Journal of Physical Chemistry C*, *118*(16), 8280–8294). Copyright (2014) American Chemical Society [66].

The fluorescence emissions of all dendrimers based on a porphyrin core were recorded and the absolute emission spectra are shown in Figure 6. If we compare the *Y*-axes on the left and on the right corresponding to the porphyrinic compounds and 1-pyrenebutanol (quantum yield of 0.52), respectively, we can realize how efficient is FRET from an excited 1-pyrenebutoxy to the ground-state of the porphyrin unit. A reduction in the fluorescence emission intensity of more than two orders of magnitude was observed.

A remarkable FRET efficiency was expected in these dendritic constructs given that the distance between the center of the porphyrin and the center of the pyrenyl unit in a fully extended conformation (*d_Por_*_−*Py*_*^EXT^*) varies between 18 and 35 Å depending on the dendritic construct. The Förster radius (R_0_) value was to equal 51.8 ± 0.2 and 48.7 ± 0.3 Å for a free-base and a Zn-metalated porphyrin, respectively (Table 2). The distance *d_Por−Py_^EXT^* included in Table 3 was calculated by means of molecular mechanics optimizations, using the program Hyperchem (Hypercube, ink., Gainesville, FL, USA). Since *d_Por–Py_^EXT^* is much smaller than R_0_ for all the dendronized porphyrins, FRET is expected to take place on a faster time scale than the formation of excimers within a Py_2_-G1OH or Py_4_-G2OH dendron.

This conclusion was verified experimentally by time-resolved fluorescence experiments. The fluorescence decays of the pyrene monomer and porphyrin in compounds Py_2_-TMEG1 and Py_4_-TMEG2 were analyzed applying the model free analysis (MFA) [66]. In these constructs an excited pyrene units transfers their excess energy so efficiently to the porphyrin core that it is deactivated before having the possibility to interact with a ground-state pyrene to give an excimer. Therefore, it can be deduced that the presence of an excimer emission band in the recorded spectra (Figure 6) for the porphyrin-cored dendritic structures is due to the small amount of impurities of pyrene derivatives. 

## 3. Dendritic Molecules Bearing Peripheral Pyrene Groups as Donors and a Fullerene as Acceptor

Recently, interest for materials based on fullerene C_60_ derivatives has increased since this type of materials has promising properties for the development of new materials for technological applications. Particularly, performances of fullerene-based compounds have been reported for solar energy conversion [68,69]. After this discovery [70], many strategies to functionalize the fullerene C_60_ were proposed [71,72,73,74,75] in order to generate new kinds of materials. The reason for this interest is due to its chemical and physical proprieties, such as for example its absorption in the UV region of the electromagnetic spectra [76]. The main drawback of the fullerene C_60_ is that it is poorly soluble in organic solvents [77,78]. Fullerene C_60_ can be synthetically modified in order to increase its solubility without affecting its photophysical and electrochemical properties [76,79]. Fullerene C_60_ derivatives possess a large variety of applications that are ranging from chemosensors [80,81,82,83] to biological probes [84,85,86] and photocatalyst [87,88,89,90]. A large number fullerene based dyads have been published, however, there are only a few reports about pyrene-fullerene C_60_ derivatives [91,92,93,94].

**Figure 7:**
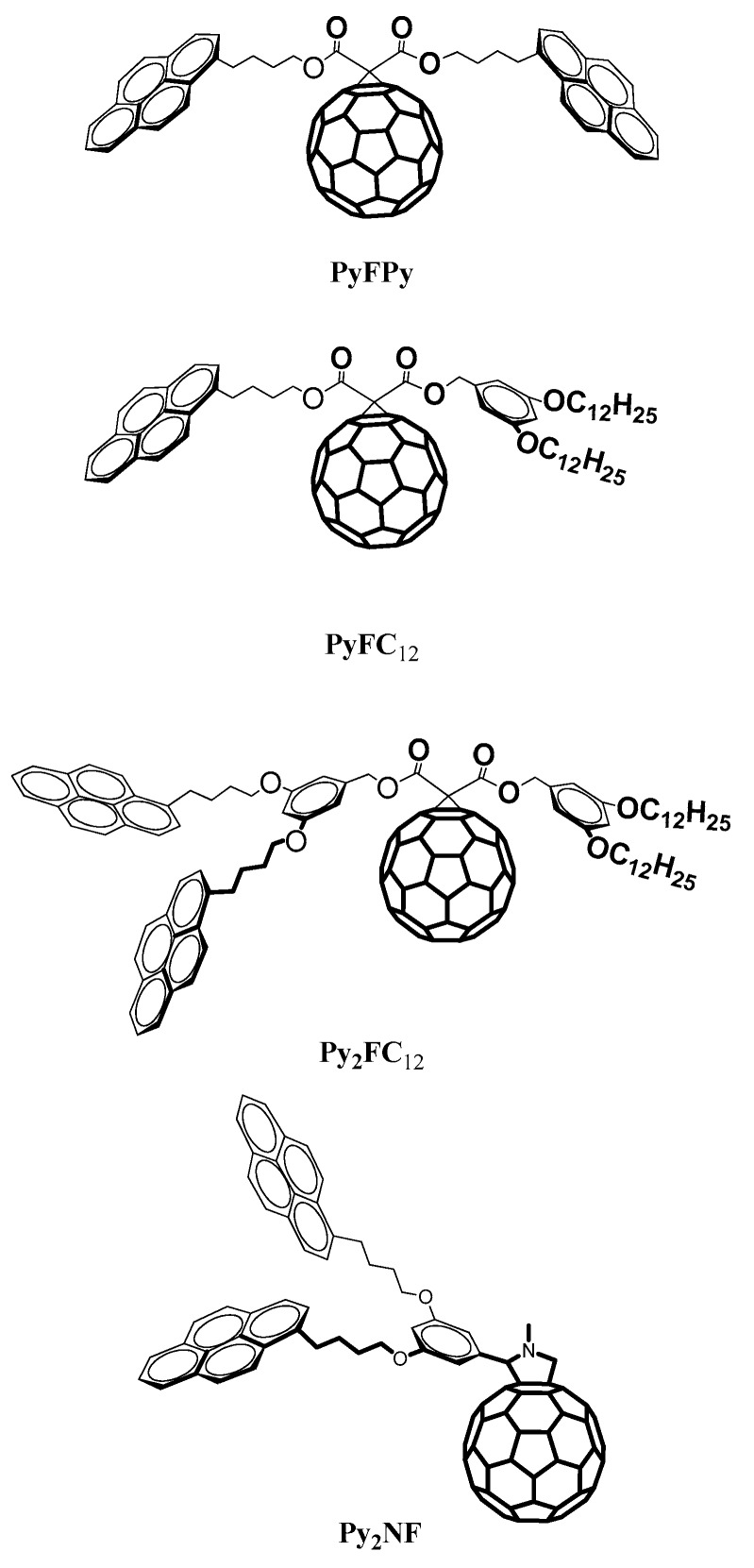
Structures of the novel compounds bearing pyrene and Fullerene C_60_ [48].

We have reported a new series of pyrene-fullerene C_60_ dyads, and we have studied their optical and photophysical properties (Figure 7) [48]. 

**Figure 8:**
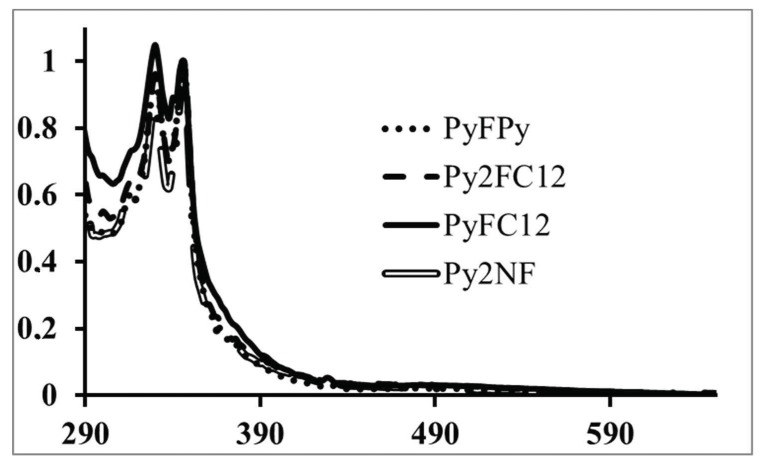
Absorption spectra of pyrene-fullerene C_60_ compounds [48].

UV–VIS analysis for the pyrene-fullerene C_60_ dyads was performed in toluene (Figure 8). It showed that the series exhibited the characteristic bands of the pyrene chromophore at 346 nm due to S_0_ → S_2_ transition. No remarkable change was observed for this transition in comparison with 1-pyrenebutanol, the compound used as model in this experiment. Moreover, for all the pyrene-fullerene C_60_ dyads the absorption band at 330 nm was more intense that the band observed in the spectra of 1-pyrenebutanol at the same wavelength and the absorption was extended in the visible region. Those two features were attributed to the fullerene C_60_ cage. In the case of PyFPy, a strong band at 330 nm followed by another intense band at 346 nm were observed. The first one was attributed to the C_60_ cage and the second one to the pyrene absorption. Contrarily, in the case of PyFC_12_ and Py_2_FC_12_ the pyrene bands are more resolved due to the higher concentration of pyrene in the molecules.

**Figure 9:**
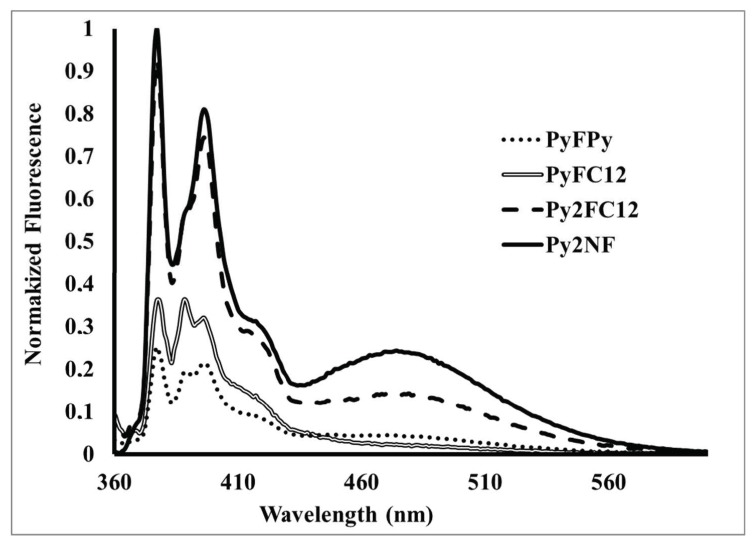
Emission spectra of pyrene-fullerene C_60_ compounds [48].

The fluorescence spectra of the compounds show the typical emission of pyrene monomer at 376 nm as illustrated in Figure 9. Only compounds PyFPy, Py_2_FC_12_ and Py2NF are able to form excimers due to the presence of two pyrene units in the structures. In the case of PyFPy, the excimer formation is blocked by the presence of the C_60_ cage that obstruct rapprochement of both pyrene units and in the spectra, there are no signal for the excimer emission (Figure 9). On the other hand, compounds Py_2_FC_12_ and Py_2_NF present the two pyrene units that are free of the fullerene steric influence and, therefore, the excimer formation is possible. This behavior is supported by the presence of the excimer band at 470 nm (Figure 9).

The relative quantum yield was calculated using 1-pyrenebutanol as standard. The obtained values are shown in Table 4. The emission of the malonate precursors that are not containing a fullerene unit was much higher than the emission of the pyrene-fullerene constructs. Low quantum yield values obtained for the pyrene-fullerene C_60_ constructs and the corresponding values of quenching corroborated that efficient FRET between pyrene and fullerene C_60_ chromophores is taking place [48].

## 4. Dendritic Molecules Bearing Peripheral Pyrene Groups as Donors and an Organometallic Complex as Acceptor 

It is of interest to include metal ions in the structure of dendrimers in order to obtain complex molecules combining the properties of both entities; the structural ordered properties from the dendritic shell and the redox properties from metal ions [95,96]. In the literature, many examples of metal ions incorporated in dendrimers have been reported with different applications, such as drug carrier, catalysts, and enzyme mimics [97,98]. In some cases, the resulting organometallic complexes are able to accept energy transfer from a donor moiety [99,100,101].

The specific case of the incorporation of ruthenium bipyridine complexes into photoactive dendrimer has been studied [102,103,104,105]. Ruthenium bypiridine complexes have attracted the attention of many researcher due to chemical stability, redox properties, as well as fluorescence emission and excited state lifetime [106]. The complexes of ruthenium with bipyridine ligands are presenting typical UV–VIS spectra. The first band observed at about 280 nm corresponds to the ligand centred (LC) transition and the second band observed in a range from 450 to 480 nm corresponds to the metal to ligand charge transfer (MLCT) band. The long-lived emission occurs from the ^3^MLCT state with low quantum yield [107,108]. Ruthenium bipyridine complexes are able to accept energy transfer from fluorescent donors through FRET [109]. Moreover, one application of this type of compound include the field of dye-sensitized solar cells [110,111]. 

Coordination complexes of ruthenium incorporating pyrene units have been reported [112]. Moreover, ruthenium bipyridine complex covalently linked to pyrene units have shown prolonged emission lifetime due to the fact that the energy levels of the ^3^MLCT state of the ruthenium complex and of the pyrene triplet state can be tuned to almost match each other. Therefore, we used our pyrene-based dendrimer to design new organometallic complexes of ruthenium. The benzylic core of our dendrimer was modified to a bipyridine core in order to obtain a suitable ligand to form ruthenium complexes [49]. 

**Figure 10:**
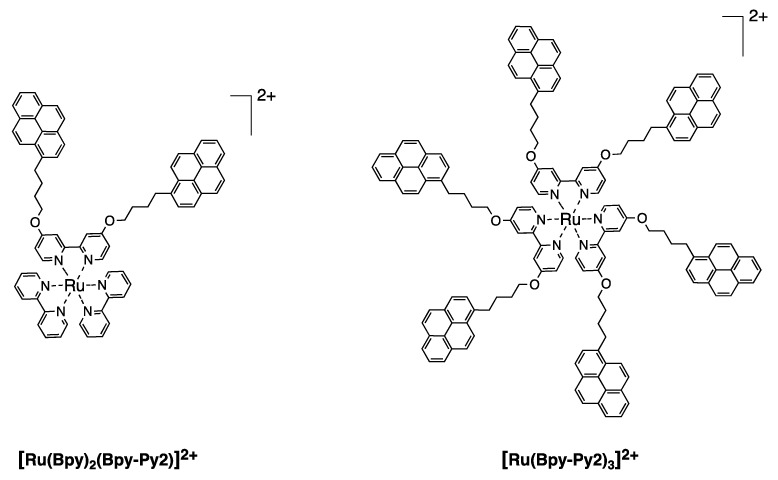
Structures of the ruthenium bipyridine complexes containing two and six pyrene units [49].

**Figure 11:**
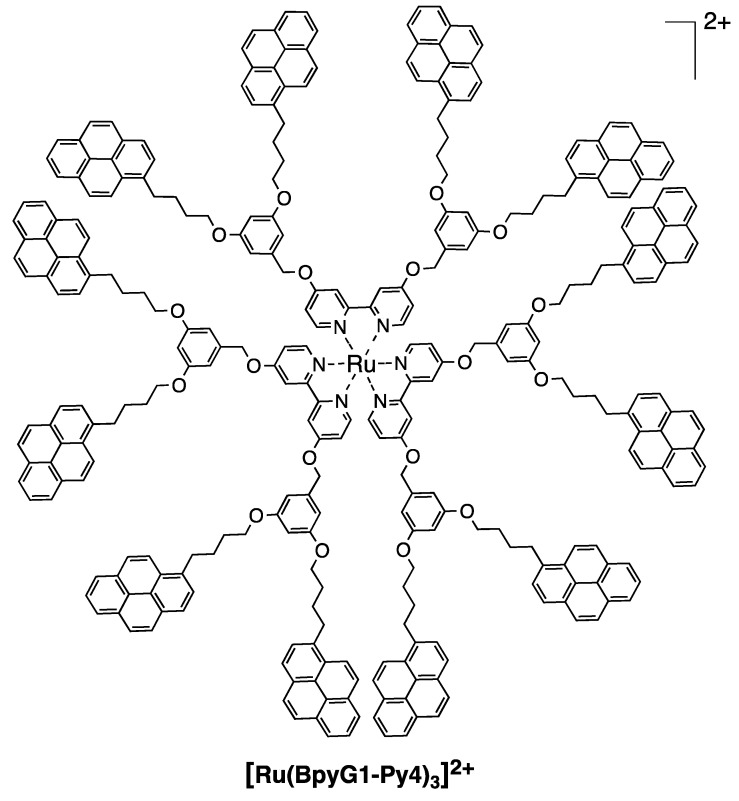
Structure of the dendritic ruthenium bipyridine complex possessing 12 pyrene units [49].

**Figure 12:**
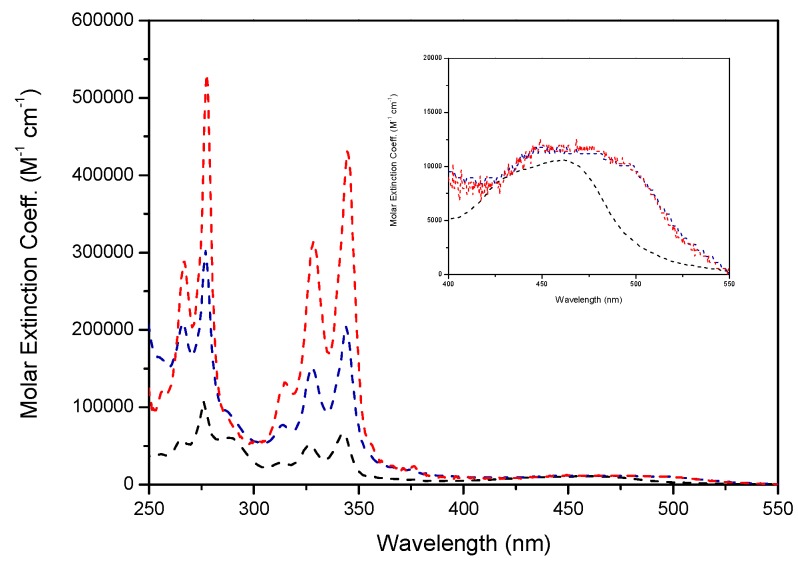
Absorption spectra of Ru(II) trisbipyridine complexes **[Ru(Bpy)_2_(Bpy-Py2)]^2+^** (black) in acetonitrile and **[Ru(Bpy-Py2)_3_]^2+^** (blue) and **[Ru(BpyG1-Py4)_3_]^2+^** (red) in THF. “Reprinted from Polymers, 99, Vonlanthen, M.; Cevallos-Vallejo, A.; Aguilar-Ortíz, E.; Ruiu, A.; Porcu, P.; Rivera, E., Synthesis, characterization and photophysical studies of novel pyrene labelled ruthenium (II) trisbipyridine complex cored dendrimers, 13–20, Copyright (2016), with permission from Elsevier” [49].

We designed and synthesized new organometallic complexes bearing 2, 6, and 12 pyrene units in the periphery and a bipyridine core (**[Ru(Bpy)_2_(Bpy-Py2)]^2+^**, **[Ru(Bpy-Py2)_3_]^2+^** and **[Ru(BpyG1-Py4)_3_]^2+^**) (Figure 10 and Figure 11). The obtained compounds showed absorption spectra that are reflecting the absorption properties of a pyrene moiety and of the corresponding bipyridine complex measured separately (Figure 12). This is an indication that, when the compounds are present in the ground state, both chromophores are not interacting. The absorption band of pyrene is observed at 344 nm for the S_0_ → S_2_ transition for the three complexes. The absorption bands of the ruthenium bipyridine complexes depend on the substitution of the bipyridine ligand. In the case of **[Ru(Bpy)_2_(Bpy-Py2)]^2+^**, the MLCT band was observed at 462 nm. A red shift to 480 nm is observed for the other two complexes **[Ru(Bpy-Py2)_3_]^2+^** and **[Ru(BpyG1-Py4)_3_]^2+^**. This is due to the stabilization effect of the oxygen atoms that are directly linked to the bipyridine moieties. The corresponding extinction coefficient are also corresponding to the respective components of the complexes. The absorption band of pyrene presents a high extinction coefficient and increases proportionally with the increasing number of pyrene units. The extinction coefficient of the MLCT transition band presents values of about 12,000 M^−1^ cm^−1^ which is comparable to the reported values for MLCT transitions of ruthenium bipyridine complexes. 

**Figure 13:**
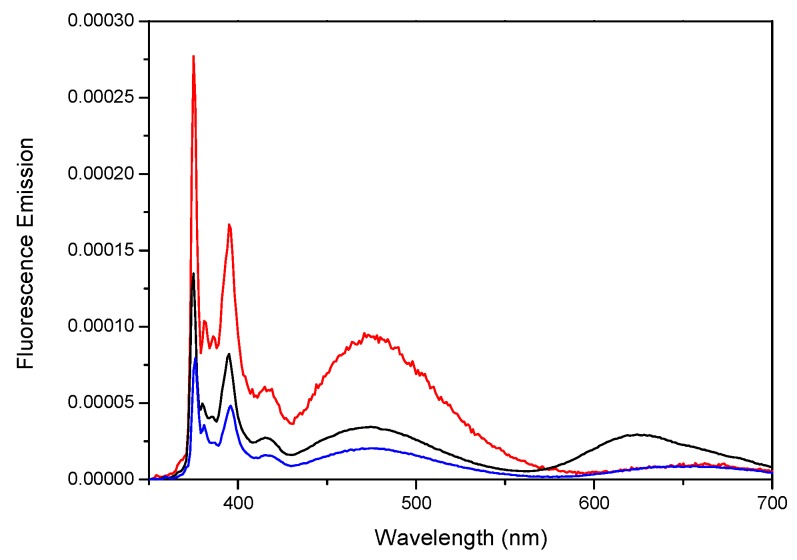
Emission spectra of the Ru(II) trisbipyridine complexes **[Ru(Bpy)_2_(Bpy-Py2)]^2+^** (black) in acetonitrile and **[Ru(Bpy-Py2)_3_]^2+^** (blue) and **[Ru(BpyG1-Py4)_3_]^2+^** (red) in THF. “Reprinted from Polymers, 99, Vonlanthen, M.; Cevallos-Vallejo, A.; Aguilar-Ortíz, E.; Ruiu, A.; Porcu, P.; Rivera, E., Synthesis, characterization and photophysical studies of novel pyrene labelled ruthenium (II) trisbipyridine complex cored dendrimers, 13–20, Copyright (2016), with permission from Elsevier” [49].

The obtained compounds present very efficient energy transfer from the pyrene moiety to the metal complex core. When they are excited at the maximum of absorption of the pyrene unit at 344 nm, weak residual emission from the pyrene units is observed but clearly quenched compared to the free bipyridine ligands. Only 2% of residual emission of pyrene was observed for **[Ru(Bpy)_2_(Bpy-Py2)]^2+^**, 1% for **[Ru(Bpy-Py2)_3_]^2+^**, and 4% for **[Ru(BpyG1-Py4)_3_]^2+^** indicating an efficient energy transfer in all cases. For the three compounds, emission is observed in the red part of the spectrum between 600 and 700 nm corresponding to the typical emission of ruthenium bipyridine complexes (Figure 13). As for the porphyrin dendrimers described in the previous section, the quenching of the fluorescence from the pyrene donor is a good indication of efficient FRET process. It is important to note that when excited at 344 nm the percentage of light absorbed by the pyrene units increases with the increasing amount of pyrene units from 91% for **[Ru(Bpy)_2_(Bpy-Py2)]^2+^** to 97 and 99%, respectively, for compounds **[Ru(Bpy-Py2)_3_]^2+^** and **[Ru(BpyG1-Py4)_3_]^2+^** (Table 5).

Furthermore, the quenching effect of oxygen was evaluated through the measurements of quantum yield for the ruthenium bipyridine complexes. When excited at 344 nm in degased solutions, quantum yields values of 0.0174, 0.0026, and 0.0031 are obtained for **[Ru(Bpy)_2_(Bpy-Py2)]^2+^**, **[Ru(Bpy-Py2)_3_]^2+^**, and **[Ru(BpyG1-Py4)_3_]^2+^**, respectively. The same measurements were performed without degasing the solutions and the following quantum yields were obtained: 0.0032, 0.0016, and 0.0025 (Table 5). 

The difference between the quantum yield in degased solution and in air-equilibrated solutions is much lower for the complex bearing three times the first generation dendron (12 pyrene units) indicating that the dendrimer branches are functioning as a protection toward oxygen quenching. For the pyrene/ruthenium bipyridine complex pair of chromophores, we have calculated that the Förster radius is in the order of 3.30 nm. As previously calculated for the pyrene-porphyrin couple the expected distance between the chromophore is within the Förster distance, confirming an efficient FRET process.

## 5. Dendritic Molecules Bearing Peripheral Pyrene Groups as Donors and Cyclen Core as Potential Ligands for Metal Ions

As mentioned above, the incorporation of different metals in photoactive dendrimers have been performed through the inclusion of porphyrin or bipyridine ligands in the structures and this has shown potential applications in sensors, catalysis, or nanomaterials for drug delivery [95,113]. Another efficient family of ligands for metal ions is the family of aza-macrocycles. Efficient chelating effects have been reported from this kind of ligand towards metal ions [114,115,116,117,118,119]. 1,4,7,10-teraazacyclododecane (cyclen) is part of this family and has exhibited a good coordination with a wide variety of metals. Moreover it has been included in a wide range of constructs for diverse applications, such as ion sensing, ion carrier, and metal diagnosis [120,121,122,123,124]. The coordination of cyclen construct containing various chromophores with lanthanides has also been reported, leading to sensitized emission from the photoactive metal ions [125,126].

Only a few reports of FRET from pyrene to lanthanide photoactive ions have been reported. Using the cyclen as core of our pyrene-based dendrimer, we could obtain a construct that is able to complex lanthanide ions and, consequently, a FRET process could be observed from pyrene to the photoactive metal ion [50].

**Figure 14:**
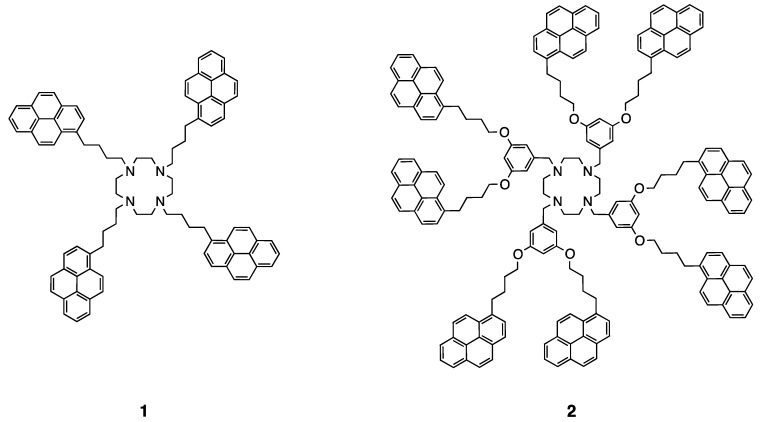
Structures of the cyclen-based ligands containing four and eight pyrene units [50].

**Figure 15:**
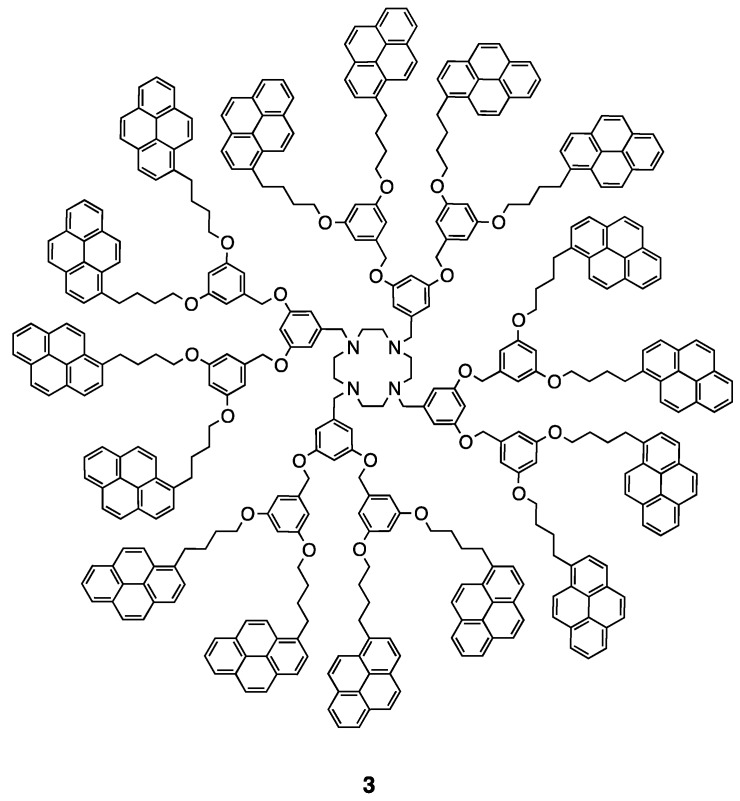
Structures of the cyclen-based ligand containing 16 pyrene units [50].

The series of dendrimers of generation zero (**1**), one (**2**) and two (**3**) bearing 4, 8, and 16 pyrene units, respectively, was synthesized (Figure 14 and Figure 15). The obtained compounds are showing the typical absorption properties of the pyrene chromophore, increasing its extinction coefficient linearly with the amount of pyrene units in the construct. The emission properties of those compounds were also studied. The amount of excimer emission in those dendrimers is increasing as expected with the increasing number of pyrene units due to the increased local concentration of pyrene [50].

**Figure 16:**
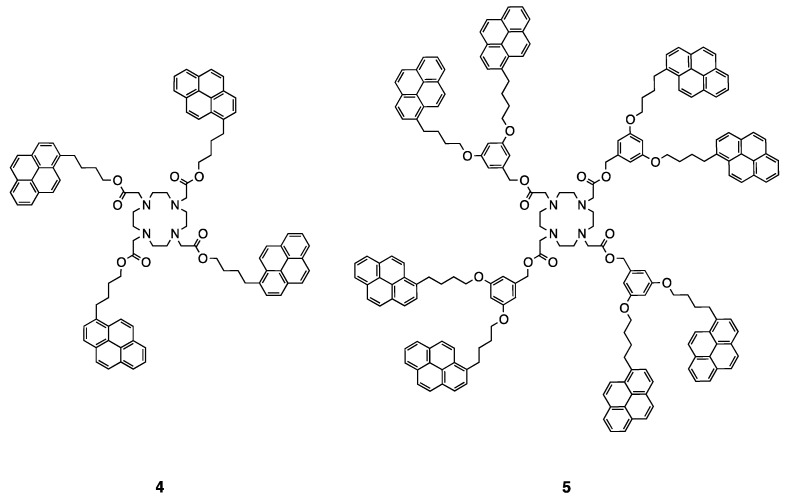
Structures of the DOTA-based ligands containing four and eight pyrene units [127].

At the time of incorporating metal ions into those dendrimers, we faced some difficulties. The ligands appeared to be less effective as foreseen towards the complexation of lanthanide ions. This may be due to the fact that the ligand has only four nitrogen atoms for the coordination of metal ions and is, therefore, a teradentate ligand. Lanthanides are known to form hexa- to octa-coordinated complexes and our ligand may not be suitable for such metal ions. Therefore, new ligands including carbonyl group linked to the cyclen core (DOTA) were designed and synthesized (**4** and **5**) (Figure 16) [127].

**Figure 17:**
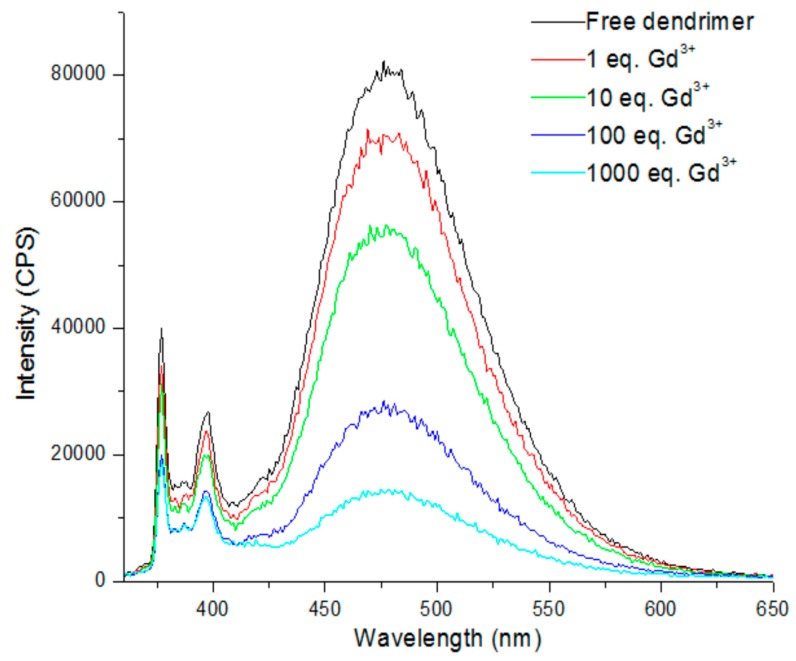
Titration of compound **4** with Gd^3+^ in chloroform [127].

The synthesis was performed and the optical properties of the obtained dendrimers were studied. The obtained photophysical characterization is very similar to that of dendrimers **1** and **2**. Titration experiments, followed by fluorescence, were carried out with compounds **4** and **5** in order to evaluate their potential for FRET studies. Sm^3+^, Eu^3+^, Gd^3+^, Tb^3+^, Er^3+^, and Zn^2+^ were tested and fluorescence quenching was observed for all metals except for Zn^2+^. Figure 17 illustrates the titration of compound **4** with Gd^3+^ [127]. This result is a primary indication that the complexes are formed and that energy transfer could occur. Chelation experiments with lanthanide ions are currently being performed in our laboratory.

## 6. Conclusions

In our interest to study pyrene dynamics in macromolecules, we have designed dendritic molecules bearing an increasing number of pyrene peripheral groups and various acceptor moieties such as porphyrin, fullerene, or ruthenium bipyridine at the core. Those molecules were expected to function as light-harvesting antennas to funnel the energy absorbed from the pyrene groups to the acceptor moiety. 

Thus, we reported that the FRET process occurs in a very efficient manner in all the designed constructs. When the pyrene moiety was excited at 344 nm, the energy was transferred to the ground state porphyrin, fullerene, or ruthenium bipyridine complex. The quenching of fluorescence of pyrene is the first indication to determine the efficiency of the FRET process. It was quantified and information about pyrene dynamics was obtained. As a result, we could determine through MF analysis that the excimer formation was much slower than the FRET phenomenon. A value of 11.2 × 10^7^ s^–1^ was found for the average rate constant of intramolecular pyrene excimer formation <k_E_>, which is much slower than the average rate constant for FRET <k_ET_> (1.8 × 10^9^ s^–1^). For this reason, we observed that the FRET process is more competitive than the excimer formation, it happens first, and the formation of excimer is precluded even in the case of higher-generation dendrons.

Dendritic structures with the donor-acceptor pair pyrene-porphyrn exhibited FRET values between 97–99% with a drastic quenching of the pyrene emission and increased emission of the porphyrinic moiety. In the case of the pyrene-fullerene C_60_ constructs, FRET efficiency values were determined in a similar range, however, unlike for the case of porphyrins, we could not observe a final emission of the fullerene C_60_ acceptor group in the UV–VIS range. On the other hand, ruthenium bipyridine complexes exhibited FRET values in the order between 96–98%, showing an emission of the acceptor group after FRET at 661 nm. Finally dendritic constructs bearing pyrene units as peripheral groups and a modified cyclen unit as the core also showed the FRET process after metallation with lanthanides, which was monitored by fluorescence titrations. The quenching of the emission of pyrene could be observed after the addition of the different lanthanide ions. 

## Figures and Tables

**Table 1:** Quantum yields and FRET efficiency for pyrene-labelled dendrons and pyrene dendronized porphyrins. Reprinted with permission from (Zaragoza-Galán, G., Fowler, M.A., Duhamel, J., Rein, R., Solladié, N., & Rivera, E. (2012). Synthesis and Characterization of Novel Pyrene-Dendronized Porphyrins Exhibiting Efficient Fluorescence Resonance Energy Transfer: Optical and Photophysical Properties. *Langmuir*, *28*(30), 11195–11205.). Copyright (2012) American Chemical Society [47].

Compound	Quantum Yield (Φ)	Quantum Yield (Φ)	E_FRET_^c^
	Pyrene Units ^a^	Porphyrin Units ^b^	
	λ_ex_ = 344 nm	λ_ex_ = 344 nm	
PyBuOH	0.52	-	-
(±error) ^d^	(0.03)		
TME	-	0.0015	-
(±error) ^d^		(0.0001)	
Py_2_-G1OH	0.63	-	-
(±error) ^d^	(0.02)		
Py_4_-G2OH	0.60	-	-
(± error) ^d^	(0.03)		
Py_2_-TMEG1	0.008	0.0015	0.99
(±error) ^d^	(0.001)	(0.00005)	
Py_4_-TMEG2	0.018	0.0014	0.97
(±error) ^d^	(0.003)	(0.0001)	

^a^ All reported fluorescence quantum yields were determined using the fluorescence quantum yield of pyrene in cyclohexane as a reference which has been reported to equal 0.32 [65]. ^b^ This value is the fluorescence quantum yield of the porphyrin core having undergone FRET from an excited pyrene to the porphyrin. It is calculated by integrating the porphyrin fluorescence intensity in Figure 3 between 580 and 680 nm, after the fluorescence spectrum was correct to account for the direct excitation of porpyrin. ^c^ E_FRET_ is the FRET efficiency, calculated using the following equation:
(1) $$E_{FRET}=1-\frac{I_{\left(Py+Por\right)}}{I_{\left(Py\right)}}$$
where *I*_(*py+por*)_ is the absolute fluorescence intensity of one mole of pyrenyl pendant in a dendron and *I_(py)_* is the absolute fluorescence intensity of one mole of pyrene attached to the corresponding dendron. ^d^ All experiments were conducted in triplicate.

**Table 2:** Förster radii (R_0_), pyrene monomer quantum yield (ϕ_Py_), and porphyrin quantum yield (ϕ_Por_) obtained by exciting the solutions at 344 nm. Reprinted with permission from (Zaragoza-Galán, G., Fowler, M., Rein, R., Solladié, N., Duhamel, J., & Rivera, E. (2014). Fluorescence Resonance Energy Transfer in Partially and Fully Labelled Pyrene Dendronized Porphyrins Studied with Model Free Analysis. *The Journal of Physical Chemistry C*, *118*(16), 8280–8294). Copyright (2014) American Chemical Society [66].

	Without Zn			With Zn		
Compound	R_0_ (Å)	ϕ_Py_ (×10^4^)	ϕ_Por_ (×10^4^)	R_0_ (Å)	ϕ_Py_ (×10^4^)	ϕ_Por_ (×10^4^)
Por-(Py_2_G1)_4_	51.4	6	15	48.3	1	27
Por-(Py_2_G1)_3_	51.8	16	15			
Por-(Py_2_G1)_2_	51.5	12	15	48.5	2	34
Por-(Py_2_G1)_1_	51.8	25	15			
Py_2_-TMEG1	52.0	13	14	48.9	12	39
Py_4_-TMEG2	52.0	18	14	49.0	23	38

**Table 3:** FRET efficiency and *d_Por_*_−*Py*_*^EXT^* for porphyrinic constructs. Reprinted with permission from (Zaragoza-Galán, G., Fowler, M., Rein, R., Solladié, N., Duhamel, J., & Rivera, E. (2014). Fluorescence Resonance Energy Transfer in Partially and Fully Labelled Pyrene Dendronized Porphyrins Studied with Model Free Analysis. *The Journal of Physical Chemistry C*, *118*(16), 8280–8294). Copyright (2014) American Chemical Society [66].

	Without Zn			With Zn		
Compound	*E*_FRET_ (SS)	*E*_FRET_ (SPC)	*d_Por–Py_^EXT^* (Å)	*E*_FRET_ (SS)	*E*_FRET_ (SPC)	*d_Por–Py_^EXT^* (Å)
Por-(Py_2_G1)_4_	0.999	1.000	18.2	1.000	1.000	17.8
Por-(Py_2_G1)_3_	0.997	1.000	18.2			17.8
Por-(Py_2_G1)_2_	0.998	1.000	18.2	1.000	0.999	17.8
Por-(Py_2_G1)_1_	0.995	1.000	18.2			17.8
Py_2_-TMEG1	0.997	0.998	30.2	0.998	0.998	29.9
Py_4_-TMEG2	0.998	0.997	34.9	0.996	0.996	34.7

**Table 4:** Relative quantum yield and FRET efficiencies of the pyrene-fullerene C_60_ compounds [48].

Compound	Relative Quantum Yield ^a^	% Quenching
1-Pyrenbutanol	1 (Donor)	-
PyFC_12_	0.01 (Dyad)	99%
PyFPy	0.01 (Dyad)	99%
Py_2_FC_12_	(Dyad)	99%
Py_2_NF	0.04 (Dyad)	96%

^a^ Measured in degassed toluene solution exciting at λ = 344 nm. Relative QY of the dyads, with respect to the donor molecules (1-pyrenebutanol and PyMPy in the range of 360 nm–560 nm.

**Table 5:** Absorption coefficients, quantum yields, and FRET efficiencies of the ruthenium-pyrene constructs “Reprinted from Polymers, 99, Vonlanthen, M.; Cevallos-Vallejo, A.; Aguilar-Ortíz, E.; Ruiu, A.; Porcu, P.; Rivera, E., Synthesis, characterization and photophysical studies of novel pyrene labelled ruthenium (II) trisbipyridine complex cored dendrimers, 13–20, Copyright (2016), with permission from Elsevier” [49].

Compound	λ_max_ abs (nm) ε(M^−1^ cm^−1^)	λ_max_ (nm)	Φ Pyrene unit λ_ex_ = 344 nm ^c^	Φ Ru (II) unit λ_ex_ = 344 nm ^d^	Φ Ru (II) unit λ_ex_ = 452 nm ^d^	E_FRET_^e^
**[Ru(Bpy)_2_**	342/69’000	625	-	0.0032 ^a^	0.0052 ^a^	0.98
**(Bpy-Py2)]^2+^**	462/10’600		0.008 ^b^	0.0174 ^b^	0.0277 ^b^	
**[Ru(Bpy-Py2)_3_]^2+^**	344/210’900	661	-	0.0016 ^a^	0.0028 ^a^	0.99
	480/11’400		0.004 ^b^	0.0026 ^b^	0.0053 ^b^	
**[Ru(BpyG1-Py4)_3_]^2+^**	344/411’000	661	-	0.0025 ^a^	0.0029 ^a^	0.96
	480/11’200		0.014 ^b^	0.0031 ^b^	0.0050 ^b^	

Fluorescence measurements were done in acetonitrile (**[Ru(Bpy)_2_(Bpy-Py2)]^2+^**) or THF (**[Ru(Bpy-Py2)_3_]^2+^** and **[Ru(BpyG1-Py4)_3_]^2+^**) at 0.1 OD at 344 nm. ^a^ Aerated solution; ^b^ Deaerated solution; ^c^ Quantum yields were determined relative to quinine sulfate (Φ = 0.546) in 0.05 M sulfuric acid for pyrene units. ^d^ Quantum yields were determined relative to [Ru(Bpy)_3_]^2+^ (Φ = 0.016) in air-equilibrated acetonitrile solution [103]. ^e^ FRET efficiencies were calculated according to the following equation: *FRET* = 1 − (I_DA_/I_D_).

## References

[1] Vögtle F., Richardt G., Werner N. (2009). Dendrimer Chemistry: Concepts, Syntheses, Properties, Applications.

[2] Tomalia D.A., Naylor A.M., Goddard W.A. (1990). Starburst Dendrimers: Molecular-Level Control of Size, Shape, Surface Chemistry, Topology, and Flexibility from Atoms to Macroscopic Matter. Angew. Chem. Int. Ed..

[3] Newkome G.R., Yao Z., Baker G.R., Gupta V.K. (1985). Micelles. Part 1. Cascade molecules: A new approach to micelles. A [27]-arborol. J. Org. Chem..

[4] Nantalaksakul A., Reddy D.R., Bardeen C.J., Thayumanavan S. (2006). Light harvesting dendrimers. Photosynth. Res..

[5] Mignani S., Rodrigues J., Tomas H., Zablocka M., Shi X., Caminade A.M., Majoral J.P. (2018). Dendrimers in combination with natural products and analogues as anti-cancer agents. Chem. Soc. Rev..

[6] Caminade A.M., Turrin C.O. (2014). Dendrimers for drug delivery. J. Mater. Chem. B.

[7] Menjoge A.R., Kannan R.M., Tomalia D.A. (2010). Dendrimer-based drug and imaging conjugates: Design considerations for nanomedical applications. Drug Discov. Today.

[8] Sun H.J., Zhang S., Percec V. (2015). From structure to function via complex supramolecular dendrimer systems. Chem. Soc. Rev..

[9] Tang Z., He C., Tian H., Ding J., Hsiao B.S., Chu B., Chen X. (2016). Polymeric nanostructured materials for biomedical applications. Prog. Polym. Sci..

[10] Wang D., Deraedt C., Ruiz J., Astruc D. (2015). Magnetic and Dendritic Catalysts. Acc. Chem. Res..

[11] Harriman A. (2015). Artificial light-harvesting arrays for solar energy conversion. Chem. Commun..

[12] Astruc D., Boisselier E., Ornelas C. (2010). Dendrimers designed for functions: From physical, photophysical, and supramolecular properties to applications in sensing, catalysis, molecular electronics, photonics, and nanomedicine. Chem. Rev..

[13] Ceroni P., Bergamini G., Marchioni F., Balzani V. (2005). Luminescence as a tool to investigate dendrimer properties. Prog. Polym. Sci..

[14] Adronov A., Fréchet J. (2000). Light-harvesting dendrimers. Chem. Commun..

[15] Fréchet J.M.J. (2003). Dendrimers and other dendritic macromolecules: From building blocks to functional assemblies in nanoscience and nanotechnology. J. Polym. Sci. Part A Polym. Chem..

[16] Balzani V., Ceroni P., Maestri M., Vicinelli V. (2003). Light-harvesting dendrimers. Curr. Opin. Chem. Biol..

[17] Zhang X., Zeng Y., Yu T., Chen J., Yang G., Li Y. (2014). Advances in photofunctional dendrimers for solar energy conversion. J. Phys. Chem. Lett..

[18] Mula S., Frein S., Russo V., Ulrich G., Ziessel R., Barberá J., Deschenaux R. (2015). Red and blue liquid-crystalline borondipyrromethene dendrimers. Chem. Mater..

[19] Lo S.C., Burn P.L. (2007). Development of dendrimers: Macromolecules for use in organic light-emitting diodes and solar cells. Chem. Rev..

[20] Satapathy R., Ramesh M., Padhy H., Chiang I.H., Chu C.W., Wei K.H., Lin H.C. (2014). Novel metallo-dendrimers containing various Ru core ligands and dendritic thiophene arms for photovoltaic applications. Polym. Chem..

[21] Krieger A., Fuenzalida Werner J.P., Mariani G., Gröhn F. (2017). Functional Supramolecular Porphyrin-Dendrimer Assemblies for Light Harvesting and Photocatalysis. Macromolecules.

[22] Winnik F. (1993). Photophysics of Preassociated Pyrenes in Aqueous Polymer Solutions and in Other Organized Media. Chem. Rev..

[23] Winnik M.A. (1985). End-to-End Cyclization of Polymer Chains. Acc. Chem. Res..

[24] Duhamel J. (2006). Polymer chain dynamics in solution probed with a fluorescence blob model. Acc. Chem. Res..

[25] Duhamel J. (2005). Pyrene florescence to study polymeric systems. Molecular Interfacial Phenomena of Polymers and Biopolymers.

[26] Duhamel J. (2012). Internal dynamics of dendritic molecules probed by pyrene excimer formation. Polymers.

[27] Duhamel J. (2012). New insights in the study of pyrene excimer fluorescence to characterize macromolecules and their supramolecular assemblies in solution. Langmuir.

[28] Figueira-Duarte T.M., Müllen K. (2011). Pyrene-based materials for organic electronics. Chem. Rev..

[29] Alqurashy B.A., Cartwright L., Iraqi A., Zhang Y., Lidzey D.G. (2017). Pyrene–benzothiadiazole-based copolymers for application in photovoltaic devices. Polym. Adv. Technol..

[30] Feng X., Hu J.Y., Redshaw C., Yamato T. (2016). Functionalization of Pyrene To Prepare Luminescent Materials—Typical Examples of Synthetic Methodology. Chem. A Eur. J..

[31] Salunke J.K., Wong F.L., Feron K., Manzhos S., Lo M.F., Shinde D., Patil A., Lee C.S., Roy V.A.L., Sonar P. (2016). Phenothiazine and carbazole substituted pyrene based electroluminescent organic semiconductors for OLED devices. J. Mater. Chem. C.

[32] Ogawa M., Momotake A., Arai T. (2004). Water-soluble poly(aryl ether) dendrimers as a potential fluorescent detergent to form micelles at very low CMC. Tetrahedron Lett..

[33] Keyes-Baig C., Duhamel J., Wettig S. (2011). Characterization of the behavior of a pyrene substituted gemini surfactant in water by fluorescence. Langmuir.

[34] Yip J., Duhamel J., Bahun G.J., Adronov A. (2010). A study of the dynamics of the branch ends of a series of pyrene-labeled dendrimers based on pyrene excimer formation. J. Phys. Chem. B.

[35] Rivera E., Aguilar-Martínez M., Terán G., Flores R.F., Bautista-Martínez J.A. (2005). Thermal, optical, electrochemical properties and conductivity of *trans*- and *cis*-poly(1-ethynylpyrene): A comparative investigation. Polymer.

[36] Illescas J., Caicedo C., Zaragoza-Galán G., Ramírez-Fuentes Y.S., Gelover-Santiago A., Rivera E. (2011). Synthesis, characterization and optical properties of novel well-defined di(1-ethynylpyrene)s. Synth. Met..

[37] Stewart G.M., Fox M.A. (1996). Chromophore-labeled dendrons as light harvesting antennae. J. Am. Chem. Soc..

[38] Cicchi S., Fabbrizzi P., Ghini G., Brandi A., Foggi P., Marcelli A., Righini R., Botta C. (2009). Pyrene-excimers-based antenna systems. Chem. A Eur. J..

[39] Vanjinathan M., Lin H.C., Nasar A.S. (2011). Synthesis, characterization and photophysical properties of DCM-based light-harvesting dendrimers. Macromol. Chem. Phys..

[40] Fogel Y., Zhi L., Rouhanipour A., Andrienko D., Räder H.J., Müllen K. (2009). Graphitic Nanoribbons with Dibenzo[e,l]pyrene Repeat Units: Synthesis and Self-Assembly. Macromolecules.

[41] Rivera E., Belletête M., Zhu X.X., Durocher G., Giasson R. (2002). Novel polyacetylenes containing pendant 1-pyrenyl groups: Synthesis, characterization, and thermal and optical properties. Polymer.

[42] Rivera E., Wang R., Zhu X.X., Zargarian D., Giasson R. (2003). Preparation of cis-poly(1-ethynylpyrene) using (1-Me-indenyl)(PPh3)Ni-C≡C-Ph/methylaluminoxane as catalyst. J. Mol. Catal. A Chem..

[43] Belletête M., Rivera E., Giasson R., Zhu X.X., Durocher G. (2004). UV-Vis and fluorescence study of polyacetylenes with pendant 1-pyrenyl groups: A comparative investigation of cis- and trans-poly(1-ethynyl-pyrene). Synth. Met..

[44] Morales-Saavedra O.G., Rivera E. (2006). Linear and nonlinear optical properties of *trans*- and *cis*-poly(1-ethynylpyrene) based sonogel hybrid materials. Polymer.

[45] Ramírez-Fuentes Y.S., Illescas J., Gelover-Santiago A., Rivera E. (2012). Luminescent polymers containing pyrenyl groups prepared by frontal polymerization of di(ethylene glycol) ethyl ether acrylate using Trigonox-23 as initiator. Mater. Chem. Phys..

[46] Illescas J., Ramírez-Fuentes Y.S., Zaragoza-Galán G., Porcu P., Mariani A., Rivera E. (2015). PEGDA-based luminescent polymers prepared by frontal polymerization. J. Polym. Sci. Part A Polym. Chem..

[47] Zaragoza-Galán G., Fowler M.A., Duhamel J., Rein R., Solladié N., Rivera E. (2012). Synthesis and characterization of novel pyrene-dendronized porphyrins exhibiting efficient fluorescence resonance energy transfer: Optical and photophysical properties. Langmuir.

[48] Zaragoza-Galán G., Ortíz-Palacios J., Valderrama B.X., Camacho-Dávila A.A., Chávez-Flores D., Ramos-Sánchez V.H., Rivera E. (2014). Pyrene-fullerene C60 dyads as light-harvesting antennas. Molecules.

[49] Vonlanthen M., Cevallos-Vallejo A., Aguilar-Ortíz E., Ruiu A., Porcu P., Rivera E. (2016). Synthesis, characterization and photophysical studies of novel pyrene labeled ruthenium (II) trisbipyridine complex cored dendrimers. Polymer.

[50] Cevallos-Vallejo A., Vonlanthen M., Porcu P., Ruiu A., Rivera E. (2017). New cyclen-cored dendrimers functionalized with pyrene: Synthesis characterization, optical and photophysical properties. Tetrahedron Lett..

[51] Bonnett R., Buckley D.G., Burrow T., Galia A.B.B., Seville B., Songca S.P. (1993). Photobactericidal materials based on porphyrins and phthalocyanines. J. Mater. Chem..

[52] Bozja J., Sherrill J., Michielsen S., Stojiljkovic I. (2003). Porphyrin-based, light-activated antimicrobial materials. J. Polym. Sci. Part A Polym. Chem..

[53] Goldberg I. (2002). Design strategies for supramolecular porphyrin-based materials. CrystEngComm.

[54] Suijkerbuijk B.M.J.M., Klein Gebbink R.J.M. (2008). Merging Porphyrins with Organometallics: Synthesis and Applications. Angew. Chem. Int. Ed..

[55] Senge M.O., Fazekas M., Notaras E.G.A., Blau W.J., Zawadzka M., Locos O.B., Ni Mhuircheartaigh E.M. (2007). Nonlinear Optical Properties of Porphyrins. Adv. Mater..

[56] Pawlicki M., Collins H.A., Denning R.G., Anderson H.L. (2009). Two-photon absorption and the design of two-photon dyes. Angew. Chem. Int. Ed..

[57] Anderson H.L. (1999). Building molecular wires from the colours of life: Conjugated porphyrin oligomers. Chem. Commun..

[58] Camps X., Dietel E., Hirsch A., Pyo S., Echegoyen L., Hackbarth S., Röder B. (1999). Globular dendrimers involving a C60core and a tetraphenyl porphyrin function. Chem. A Eur. J..

[59] Flamigni L., Talarico A.M., Ventura B., Rein R., Solladié N. (2006). A versatile bis-porphyrin tweezer host for the assembly of noncovalent photoactive architectures: A photophysical characterization of the tweezers and their association with porphyrins and other guests. Chem. A Eur. J..

[60] Bell T.D.M., Bhosale S.V., Ghiggino K.P., Langford S.J., Woodward C.P. (2009). Synthesis and photophysical properties of a conformationally flexible mixed porphyrin star-pentamer. Aust. J. Chem..

[61] Solladie N., Hamel A., Gross M. (2000). Synthesis of multiporphyrinic α-polypeptides: Towards the study of the migration of an excited state for the mimicking of the natural light harvesting device. Tetrahedron Lett..

[62] Zhu M., Lu Y., Du Y., Li J., Wang X., Yang P. (2011). Photocatalytic hydrogen evolution without an electron mediator using a porphyrin-pyrene conjugate functionalized Pt nanocomposite as a photocatalyst. Int. J. Hydrogen Energy.

[63] Kim J.B., Leonard J.J., Longo F.R. (1972). Mechanistic study of the synthesis and spectral properties of meso-tetraarylporphyrins. J. Am. Chem. Soc..

[64] Quimby D.J., Longo F.R. (1975). Luminescence Studies on Several Tetraarylporphins and Their Zinc Derivatives. J. Am. Chem. Soc..

[65] Berlman I.B. (1971). Handbook of Fluorescence Spectra of Aromatic Molecules.

[66] Zaragoza-Galán G., Fowler M., Rein R., Solladié N., Duhamel J., Rivera E. (2014). Fluorescence resonance energy transfer in partially and fully labeled pyrene dendronized porphyrins studied with model free analysis. J. Phys. Chem. C.

[67] Galván-Miranda E.K., Zaragoza-Galán G., Rivera E., Aguilar-Martínez M., Macías-Ruvalcaba N.A. (2014). Electrochemical and spectroelectrochemical study of A4and A2B2pyrene dendronized porphyrins. Electrochim. Acta.

[68] Giacalone F., Martín N. (2006). Fullerene polymers: Synthesis and properties. Chem. Rev..

[69] Lin Y., Wang J., Zhang Z.-G., Bai H., Li Y., Zhu D., Zhan X. (2015). An Electron Acceptor Challenging Fullerenes for Efficient Polymer Solar Cells. Adv. Mater..

[70] Kroto H.W., Heath J.R., O’Brien S.C., Curl R.F., Smalley R.E. (1985). C60: Buckminsterfullerene. Nature.

[71] Chiang L.Y., Upasani R.B., Swirczewski J.W. (1992). Versatile Nitronium Chemistry for C60 Fullerene Functionalization. J. Am. Chem. Soc..

[72] Mikami K., Matsumoto S., Tonoi T., Okubo Y., Suenobu T., Fukuzumi S. (1998). Solid state photochemistry for fullerene functionalization: Solid state photoinduced electron transfer in the diels-alder reaction with anthracenes. Tetrahedron Lett..

[73] Itami K. (2011). Molecular catalysis for fullerene functionalization. Chem. Rec..

[74] Wang H.H., Schlueter J.A., Cooper A.C., Smart J.L., Whitten M.E., Geiser U., Carlson K.D., Williams J.M., Welp U., Dudek J.D. (1993). Fullerene derivatives and fullerene superconductors. J. Phys. Chem. Solids.

[75] Ball Z.T., Sivula K., Fréchet J.M.J. (2006). Well-defined fullerene-containing homopolymers and diblock copolymers with high fullerene content and their use for solution-phase and bulk organization. Macromolecules.

[76] Guldi D.M., Prato M. (2000). Excited-state properties of C60 fullerene derivatives. Acc. Chem. Res..

[77] Semenov K.N., Charykov N.A., Keskinov V.A., Piartman A.K., Blokhin A.A., Kopyrin A.A. (2010). Solubility of light fullerenes in organic solvents. J. Chem. Eng. Data.

[78] Marcus Y., Smith A.L., Korobov M.V., Mirakyan A.L., Avramenko N.V., Stukalin E.B. (2001). Solubility of C 60 Fullerene. J. Phys. Chem. B.

[79] Backer S.A., Sivula K., Kavulak D.F., Frechet J.M.J. (2007). High efficiency organic photovoltaics incorporating a new family of soluble fullerene derivatives. Chem. Mater..

[80] Sharma P.S., Dabrowski M., Noworyta K., Huynh T.P., KC C.B., Sobczak J.W., Pieta P., D’Souza F., Kutner W. (2014). Fullerene derived molecularly imprinted polymer for chemosensing of adenosine-5′-triphosphate (ATP). Anal. Chim. Acta.

[81] Haino T., Araki H., Fujiwara Y., Tanimoto Y., Fukazawa Y. (2002). Fullerene sensors based on calix[5]arene. Chem. Commun..

[82] Ciotta E., Paoloni S., Richetta M., Prosposito P., Tagliatesta P., Lorecchio C., Venditti I., Fratoddi I., Casciardi S., Pizzoferrato R. (2017). Sensitivity to heavy-metal ions of unfolded fullerene quantum dots. Sensors.

[83] Nalwa K.S., Cai Y., Thoeming A.L., Shinar J., Shinar R., Chaudhary S. (2010). Polythiophene-fullerene based photodetectors: Tuning of spectral esponse and application in photoluminescence based (Bio)chemical sensors. Adv. Mater..

[84] Nakamura S., Mashino T. (2009). Biological activities of water-soluble fullerene derivatives. J. Phys. Conf. Ser..

[85] Bosi S., Da Ros T., Spalluto G., Prato M. (2003). Fullerene derivatives: An attractive tool for biological applications. Eur. J. Med. Chem..

[86] Gibson D.G., Glass J.I., Lartigue C., Noskov V.N., Chuang R.Y., Algire M.A., Benders G.A., Montague M.G., Ma L., Moodie M.M. (2010). Creation of a bacterial cell controlled by a chemically synthesized genome. Science.

[87] Luo C.-Y., Huang W.-Q., Xu L., Yang Y.-C., Li X., Hu W., Peng P., Huang G.-F. (2016). Enhanced photocatalytic performance of an Ag3PO4 photocatalyst via fullerene modification: First-principles study. Phys. Chem. Chem. Phys..

[88] Mengele A.K., Kaufhold S., Streb C., Rau S. (2016). Generation of a stable supramolecular hydrogen evolving photocatalyst by alteration of the catalytic center. Dalton Trans..

[89] Cho E.-C., Ciou J.-H., Zheng J.-H., Pan J., Hsiao Y.-S., Lee K.-C., Huang J.-H. (2015). Fullerene C70 decorated TiO2 nanowires for visible-light-responsive photocatalyst. Appl. Surf. Sci..

[90] Apostolopoulou V., Vakros J., Kordulis C., Lycourghiotis A. (2009). Preparation and characterization of [60] fullerene nanoparticles supported on titania used as a photocatalyst. Colloids Surf. A Physicochem. Eng. Asp..

[91] Cominetti A., Pellegrino A., Longo L., Po R., Tacca A., Carbonera C., Salvalaggio M., Baldrighi M., Meille S.V. (2015). Polymer solar cells based on poly(3-hexylthiophene) and fullerene: Pyrene acceptor systems. Mater. Chem. Phys..

[92] Matsuo Y., Morita K., Nakamura E. (2008). Penta(pyrenyl)[60]fullerenes: Pyrene-pyrene and [60]fullerene-pyrene interactions in the crystal and in solution. Chem. Asian J..

[93] Sandanayaka A.S.D., Araki Y., Ito O., Deviprasad G.R., Smith P.M., Rogers L.M., Zandler M.E., D’Souza F. (2006). Photoinduced electron transfer in fullerene triads bearing pyrene and fluorene. Chem. Phys..

[94] Hwang Y.L., Hwang K.C. (1999). Nonlinear Stern-Volmer fluorescence quenching of pyrene by C60/70. Fuller Sci. Technol..

[95] Newkome G.R., He E., Moorefield C.N. (1999). Suprasupermolecules with Novel Properties: Metallodendrimers. Chem. Rev..

[96] Astruc D., Ruiz J. (2015). The Redox Functions of Metallodendrimers. J. Inorg. Organomet. Polym. Mater..

[97] Méry D., Astruc D. (2006). Dendritic catalysis: Major concepts and recent progress. Coord. Chem. Rev..

[98] Albrecht M., Hovestad N.J., Boersma J., Van Koten G. (2001). Multiple use of soluble metallodendritic materials as catalysts and dyes. Chem. A Eur. J..

[99] D’Ambruoso G.D., McGrath D.V. (2008). Energy harvesting in synthetic dendrimer materials. Adv. Polym. Sci..

[100] Balzani V., Campagna S., Denti G., Juris A., Serroni S., Venturi M. (1998). Designing Dendrimers Based on Transition-Metal Complexes. Light-Harvesting Properties and Predetermined Redox Patterns. Acc. Chem. Res..

[101] Larsen J., Puntoriero F., Pascher T., McClenaghan N., Campagna S., Åkesson E., Sundström V. (2007). Extending the light-harvesting properties of transition-metal dendrimers. ChemPhysChem.

[102] Vögtle F., Plevoets M., Nieger M., Azzellini G.C., Credi A., De Cola L., De Marchis V., Venturi M., Balzani V. (1999). Dendrimers with a photoactive and redox-active [Ru(bpy)3]2+-type core: Photophysical properties, electrochemical behavior, and excited-state electron-transfer reactions. J. Am. Chem. Soc..

[103] Issberner J., Vogtle F., De Cola L., Balzani V. (1997). Dendritic bipyridine ligands and their tris(bipyridine)ruthenium(II) chelates—Syntheses, absorption spectra, and photophysical properties. Chem. A Eur. J..

[104] Kimura M., Shiba T., Muto T., Hanabusa K., Shirai H. (2000). Energy transfer within ruthenium-cored rigid metallodendrimers. Tetrahedron Lett..

[105] Tyson D.S., Luman C.R., Castellano F.N. (2002). Photodriven electron and energy transfer from a light-harvesting metallodendrimer. Inorg. Chem..

[106] Juris A., Balzani V., Barigelletti F., Campagna S., Belser P., von Zelewsky A. (1988). Ru(II) polypyridine complexes: Photophysics, photochemistry, eletrochemistry, and chemiluminescence. Coord. Chem. Rev..

[107] Crosby G.A. (1975). Spectroscopic Investigations of Excited States of Transition-Metal Complexes. Acc. Chem. Res..

[108] Crosby G.A., Elfring W.H. (1976). Excited states of mixed ligand chelates of ruthenium(II) and rhodium(III). J. Phys. Chem..

[109] Tyson D.S., Gryczynski I., Castellano F.N. (2000). Long-Range Resonance Energy Transfer to [Ru(bpy)_3_]^2+^. J. Phys. Chem. A.

[110] Nazeeruddin M.K., Klein C., Liska P., Grätzel M. (2005). Synthesis of novel ruthenium sensitizers and their application in dye-sensitized solar cells. Coord. Chem. Rev..

[111] Hagfeldt A., Boschloo G., Sun L., Kloo L., Pettersson H. (2010). Dye-sensitized solar cells. Chem. Rev..

[112] Howarth A.J., Majewski M.B., Wolf M.O. (2015). Photophysical properties and applications of coordination complexes incorporating pyrene. Coord. Chem. Rev..

[113] Balzani V., Juris A. (2001). Photochemistry and photophysics of Ru(II)-polypyridine complexes in the Bologna group. From early studies to recent developments. Coord. Chem. Rev..

[114] Le Goff A., Gorgy K., Holzinger M., Haddad R., Zimmerman M., Cosnier S. (2011). Tris(bispyrene-bipyridine)iron(II): A supramolecular bridge for the biofunctionalization of carbon nanotubes via π-stacking and pyrene/β-cyclodextrin host-guest interactions. Chem. A Eur. J..

[115] Pedersen C.J. (1967). Cyclic Polyethers and Their Complexes with Metal Salts. J. Am. Chem. Soc..

[116] Delgado R., Félix V., Lima L.M.P., Price D.W. (2007). Metal complexes of cyclen and cyclam derivatives useful for medical applications: A discussion based on thermodynamic stability constants and structural data. Dalton Trans..

[117] Austin C.A., Chen Y., Rodgers M.T. (2012). Alkali metal cation-cyclen complexes: Effects of alkali metal cation size on the structure and binding energy. Int. J. Mass Spectrom..

[118] Gunnlaugsson T., Leonard J.P., Mulready S., Nieuwenhuyzen M. (2004). Three step vs one pot synthesis and X-ray crystallographic investigation of heptadentate triamide cyclen (1,4,7,10-tetraazacyclododecane) based ligands and some of their lanthanide ion complexes. Tetrahedron.

[119] Aime S., Botta M., Garda Z., Kucera B.E., Tircso G., Young V.G., Woods M. (2011). Properties, solution state behavior, and crystal structures of chelates of DOTMA. Inorg. Chem..

[120] Xu Z., Yoon J., Spring D.R. (2010). Fluorescent chemosensors for Zn^2+^. Chem. Soc. Rev..

[121] Bhuyan M., Katayev E., Stadlbauer S., Nonaka H., Ojida A., Hamachi I., König B. (2011). Rigid luminescent bis-zinc(II)-bis-cyclen complexes for the detection of phosphate anions and non-covalent protein labeling in aqueous solution. Eur. J. Org. Chem..

[122] Plush S.E., Gunnlaugsson T. (2007). Luminescent sensing of dicarboxylates in water by a bismacrocyclic dinuclear Eu(III) conjugate. Org. Lett..

[123] Gunnlaugsson T., Leonard J.P. (2005). Responsive lanthanide luminescent cyclen complexes: From switching/sensing to supramolecular architectures. Chem. Commun..

[124] Kimura E., Aoki S., Koike T., Shiro M. (1997). A tris(Zn(II)-1,4,7,10-tetraazacyclododecane) complex as a new receptor for phosphate dianions in aqueous solution. J. Am. Chem. Soc..

[125] Borbas K.E., Bruce J.I. (2007). Synthesis of asymmetrically substituted cyclen-based ligands for the controlled sensitisation of lanthanides. Org. Biomol. Chem..

[126] Pillai Z.S., Ceroni P., Kubeil M., Heldt J.M., Stephan H., Bergamini G. (2013). Dendrimers as Nd^3+^ ligands: Effect of generation on the efficiency of the sensitized lanthanide emission. Chem. Asian J..

[127] González Ortega J.C. (2017). Síntesis Y Caracterización De Nuevos Sistemas Dendriticos Que Contienen Grupos Donador-Aceptor Pireno-Cicleno Metalado: Estudio De Las Propiedades Ópticas Y Fotofisicas. Master’s Thesis.

